# Cultivation of Entrepreneurial Psychology and Innovation Ability by New Media Art Under the Reform of Publishing Industry

**DOI:** 10.3389/fpsyg.2021.725749

**Published:** 2021-11-01

**Authors:** Mingjie Zhang, Fangbin Song

**Affiliations:** ^1^School of Information Management, Nanjing University, Nanjing, China; ^2^School of Design Art & Media, Nanjing University of Science and Technology, Nanjing, China

**Keywords:** new media art, Yangtze River Delta region, reform of the publishing industry, digital transformation, entrepreneurial psychology, innovation capacity

## Abstract

In order to optimize the resource allocation of the traditional publishing industry in the new media era, it is proposed to integrate the traditional publishing and digital publishing industries to solve the problem of unbalanced resource distribution under dual-track conditions. Professional talents with innovative entrepreneurial ability and psychology in colleges and universities are cultivated to promote the integration and reform process of the publishing industry under the background of new media art. First, the study analyzes the digital reform issues facing the development of the publishing industry in the new media era. Second, in view of the development situation of the publishing industry in the Yangtze River Delta, it is proposed to establish a development model of integrated publishing in the Yangtze River Delta through resource allocation. Then, under the new media art form, the teaching mode of creative and entrepreneurial talents training in art colleges and universities is optimized to cultivate students’ innovative ability and entrepreneurial positive psychology. The research results show that the number of books printed in Shanghai in the Yangtze River Delta is 13,000 types per year, and the number is still rising; however, periodicals and newspapers are affected by the new media industry, and the number of publications is declining. The printing volume has dropped by 50% in 9years; the questionnaire survey results show that 68% of the students are very interested in entrepreneurial activities, but 53% of the students have not carried out entrepreneurial activities at all, indicating that the students’ entrepreneurial ability is insufficient. The results provide a reference for studying the reform direction of the publishing industry and cultivating entrepreneurial talents in the context of new media.

## Introduction

With the continuous development of science and technology, knowledge is refreshed faster and faster, which makes the demand for the timeliness of information become increasing. In this case, the traditional publishing industry encounters heavy pressure due to its long cycle and high cost. The traditional publishing industry uses paper media like newspapers and books as the carrier for information transmission, and this is difficult to timely and effectively transmit and integrate information (Boratyńska, 2019; Fan, 2019; Magadán-Díaz and Rivas-García, 2021). With the development of digital technology, paper media is integrating with digital media, which makes the publishing industry develop to some extent. And media integration also provides an effective information transmission carrier for the publishing industry, meets people’s actual demand for information, and helps the traditional publishing industry develop better in the process of media convergence by various new technologies (Have and Pedersen, 2020). New media, with the electronic media as the basic language of the new art form, is based on digital technology and penetrates all sectors of society. The new media art, produced in the digital process, provides a powerful means of innovation and entrepreneurship for entrepreneurs of related people, changing the way of information dissemination through the integration of information, and improving the reading experience of readers (Girija, 2020). Therefore, the integration of the traditional publishing industry and the digital publishing industry is an irresistible general trend of the information age. And it provides a strong innovation and entrepreneurship environment for entrepreneurs by the optimization of the resource allocation of the traditional publishing and digital publishing industry.

Scholars in China and foreign countries have studied the changes and development of the art industry under the background of digitalization. Çöteli (2019) pointed out that culture reflects all values retained by society. In the era of information, culture has been digitized. The reason is that individuals are presented as digital identities in their life, and this phenomenon is enhanced in the media of digital communication, resulting in significant changes in cultural concepts. Culture in real life is guided by digital culture. Wibisono and Koesrindartoto (2020) studied the competition among publishing companies under technological and social changes and proposed that publishing companies need to develop effective business strategies to enhance their competitiveness and sustainability in the publishing industry. The study found that lowing costs were the most suitable business strategy. And establishing the developed production and distribution mode can increase the profits of publishing enterprises. Wei et al. (2019) studied the mediating effect of remediation skills and entrepreneurship on entrepreneurship education and innovation and used structural equations to analyze the collected data. The results showed that there was a positive correlation between entrepreneurship education and innovation. Political skills and entrepreneurial opportunities mediated the relationship between entrepreneurship education and innovation. Girija (2019) studied the impact of increased digital device users on digital media entrepreneurship in the context of Internet penetration. It was found that in the media environment controlled by enterprises, digital news was vulnerable to commercial and political pressure, while start-ups of new media claimed to take public services as the purpose. In the analysis, it was found that news start-ups relied on the support of enterprise funds and technology, and it was difficult to maintain long-term development without public funds and government support. Wu and Wu (2017) studied the status of entrepreneurship education in the Asia-Pacific region and put forward innovative ideas. This study provides a reference for establishing an entrepreneurship education system from functional, personality, and behavioral perspectives.

Under the form of new media, the development of the traditional publishing industry has been greatly affected. In order to better promote the development of the publishing industry, it is necessary to further promote the digital transformation of the traditional publishing industry. Firstly, the study analyzes the problems encountered in the development of the traditional publishing industry under new media art and the necessity of digital transformation. And in view of the economic characteristics and development requirements of the Yangtze River Delta region, it discusses the establishment of the Yangtze River Delta integrated publishing development model. Secondly, under the new media art form, the opportunities and psychological qualities of art and design students’ entrepreneurship are analyzed, and the innovation ability and entrepreneurial ability of students are improved by optimizing the curriculum system. The innovative point lies in: analyze the development situation of the publishing industry under new media, to judge the possibility of entrepreneurship by students in relevant colleges and universities, and to cultivate students’ entrepreneurial psychology and innovative entrepreneurial abilities by optimizing the college curriculum system. The improvement of college students’ innovation and entrepreneurship ability can better solve the difficult employment situation. Currently, the problem of difficult employment of college students has long been prominent. The reason is that higher education focuses on quantity rather than quality, and China’s economic development emphasizes growth rather than quality. To solve the problem of difficult employment, it is urgent to start from the root, which also coincides with the goal behind China’s emphasis on innovation and entrepreneurship development. The cultivation of innovative and entrepreneurial talents has played a positive role in solving the employment problem of college students.

## Development of Publishing Industry and the Cultivation of Entrepreneurial Talents Under the New Media

### Development Direction of the Traditional Publishing Industry Under New Media Art

With the development of the global economy and informationization, China’s publishing industry is experiencing the difficulties of enterprise transformation and digital transformation. The digital transformation of the traditional publishing industry includes the digitalization of the creation, edition, processing, printing, and reproduction (Magadán-Díaz and Rivas-García, 2019; Watson, 2019). As a part of China’s cultural industry, the publishing industry has two attributes of economy and society. Therefore, in the process of integrated development of the publishing industry, it is indispensable to support the resource allocation of the publishing industry, government subjects, and social forces. Promoting traditional publishing and emerging publishing is an effective way to integrate the publishing resources and production factors, which is the economic objective for the integrated development of China’s publishing industry. And in the information age, information, technology and management, and other intellectual production are important production elements, and the publishing industry is changing in the production process and consumer market resources, which have different characteristics in the process of integration, showing a diversified leading development direction in the integration of the publishing industry (Feng and Chen, 2020; Magadán-Díaz and Rivas-García, 2020a).

At present, China is accelerating the development of cultural industry and optimizing the industrial layout, forming an intensive, professional, and large-scale cultural industry development trend (Magadán-Díaz and Rivas-García, 2020b). The use of Internet technology makes new media technology an important tool for information dissemination in social culture. New media have an important impact on the channels of economic and cultural communication. More and more college students begin to use new media to establish their entrepreneurial platforms. And the entrepreneurship education of art students has become an important factor in the continuation and development of the cultural industry. The traditional publishing industry and digital publishing occupy content resources, editing resources, market resources, and policy resources in the market. Traditional publishing usually takes up content resources, editing resources, and policy resources, while digital publishing occupies technical resources and channel resources (Ritter and Pedersen, 2020). The integration development of traditional publishing and digital publishing is a resource allocation optimization process under the dual-track effect, and conflicts may occur due to uneven resource allocation in the integration process.

The integrated development of the traditional publishing industry and digital publishing industry is the requirement of the information age. The state issues a series of preferential policies and support measures for the development of the publishing industry, which provides a strong guarantee for the integrated development of the publishing industry. In the process of integration, entrepreneurs can compete for publishing resources through science and technology and publishing houses, the core of information communication in the traditional era. The traditional publishing industry processes the production and management of industrial content. In the new media era, content service and operation are more important. The result of the integration of publications can make the boundary of the two more clear and the distribution of publishing resources balanced (Toscher, 2019). The digital transformation of China’s traditional publishing industry can be divided into two stages: (1) Technology providers are dominant while content providers are passive; (2) content providers strive for dominance and technology providers occupy the market. In the future, the two will complement each other’s advantages, realize benefit-sharing, and jointly seek stable development.

The publishing industry is affected by industrial policies of regional economic development and culture, and it has obvious regional characteristics. The Yangtze River Delta region is in the leading position in China’s economic development, which makes its regional publishing industry have more development opportunities. However, there are also the following obvious defects (Sandberg et al., 2020). Under the new media, the Yangtze River Delta region tries to build a development model of integrated publishing in the Yangtze River Delta and jointly promote the open cooperation and reciprocal linkage of the publishing industry in the Yangtze River Delta. Yangtze River Delta region has a profound cultural heritage, and superior natural conditions and geographical environment, becoming an important base for the development of the modern cultural industry. Shanghai, as the economic leader in the Yangtze River Delta region, is the center of China’s publishing industry. Therefore, the publishing industry in the Yangtze River Delta is at the forefront of the country in terms of physical reform and technical personnel reserve. Meanwhile, it can promote the development of the publishing industry in Jiangsu, Zhejiang, and Huizhou (Toscher et al., 2020). At present, the Yangtze River Delta region has become a national publishing industry base with complete publishing categories, diverse varieties, and advanced technology. The development model of three provinces and one city effectively integrates the publishing industry resources in the Yangtze River Delta region, so that the region has a sound production factor of the publishing industry, fully meets the needs of regional integration development, and lays the foundation for building an integrated publishing development market in the Yangtze River Delta (Augustyn, 2020).

### Development Mode of the New Media Art Publishing Industry in Yangtze River Delta Under Resource Allocation

Due to the low degree of integration of the publishing industry in the Yangtze River Delta and the lack of corresponding coordination measures, the homogenization of the publishing industry is serious, the competitiveness in the industry is not strong, and there appear market segmentation and malicious competition. And the Yangtze River Delta region lacks complementary resources of the publishing industry, resulting in a small cooperation space, difficulties in establishing a win-win model of a large-scale business, and the phenomenon that the publishing industry market is occupied by digital publishing (Irfanullah, 2021). In addition, the publishing groups in the region are doing their own business, and their information docking is poor and unable to exchange information in the short term. And due to regional policy constraints, foreign currency cannot invest in the Yangtze River Delta region. In this case, the market share is losing with the impact of digital publishing, which harms the sustainable development of the publishing industry in the Yangtze River Delta. With the continuous development of new media, the traditional publishing industry in the Yangtze River Delta needs to integrate resources and media, adapt to the needs of the development of the times, and enhance their development strength (Cele and Wale, 2020).

In the traditional publishing industry, newspapers and books are paper media, and they are impossible to integrate information timely and effectively. Therefore, in the process of digital development, the information carrier of the publishing industry should be changed not only through the text, but also the audio and video to realize information integration so that the information can be carried by more people to meet people’s needs for information. At the same time, in the process of the integration of the new media and the publishing industry, it needs to innovate relevant technologies to help the traditional publishing industry develop and improve its services. In the context of new media art, the publishing industry is also constantly innovating. The information can be integrated by using images, videos, and audio to change the reading and communication mode of traditional publishing journals and adapt to the rapid economic development of society and people’s demand for information, with the core content of the publishing industry unchanged.

In the market economy, production factors are used not only in various production links but also in the circulation and exchange, and they have distribution abilities, which make other owners use their production factors to maximize the allocation of production factors. Therefore, the market economic system is an economic system in which production factors participate in the production, circulation, and distribution processes. Under China’s dual-track economic system, resource allocation is unbalanced. Thus, the publishing industry must be integrated to adapt to the production economy and alleviate regional cultural conflicts. In the context of informatization, innovative technologies play important roles in the integration process and ensure the innovative development of the publishing industry (Fang et al., 2019). Under the support of the current national policy, they provide an economic and political basis for the integrated development of the publishing industry. The technological revolution of new science and technology has brought great market technical support for the innovation and rise of the publishing industry. The integrated development of the publishing industry is gradually in progress.

### Influence of New Media Art on Entrepreneurs’ Psychology and Cultivation of Innovation Ability

The new media art refers to the works created by using digital art, computer graphics, network art, sound art, 3D printing, and virtual art, and other new media technologies. Compared with the old visual arts such as traditional painting and sculpture, the cultural objects and social art produced by the new media art have more obvious important characteristics of contemporary art (Ferreira et al., 2020; Hwang et al., 2020). The development of the new media transforms human thoughts into the digital era. Digital information exchange and online social networking become an important part of people’s lives. Under the guidance of digitalization, public space collapses, and people have formed a specific community. In the process of this digital revolution, the structure of the group replaces the existing rights and ruling relations, and the formed group becomes a digital group, which shows different characteristics and group structure from the general public (Wei et al., 2020). The digital group is composed of the individual, but its characteristics cannot be traced back to the individual, so the digital group does not have a soul and thought. The publishing industry is creative. Under the impact of new media art, publishing content becomes an important foundation for victory. The innovation of content, technology, and form is the basis for promoting the development of the publishing industry (Cai et al., 2019).

The integration of the traditional publishing industry and the digital publishing industry under the new media environment has higher requirements for the cultivation of innovative and entrepreneurial teaching of college students majoring in art design. Colleges and universities not only need to teach students to master the corresponding professional skills, but also need to cultivate students’ innovative ability, entrepreneurial practice ability, and entrepreneurial psychology (Sirén et al., 2020). Entrepreneurship education in colleges and universities needs to cultivate innovative talents and pays attention to the cultivation of entrepreneurs’ innovation and entrepreneurial ability. With more research on entrepreneurship education, entrepreneurial psychology has gradually become the focus of the research on the quality of entrepreneurial isolation. The entrepreneurial cognition theory believes that in the process of entrepreneurship, entrepreneurs will evaluate entrepreneurial opportunities and carry out a series of risk-taking activities and make certain considerations and decisions (Genc et al., 2019). College students’ entrepreneurial psychological quality is a stable psychological characteristic of innovation generated by the interaction of environment and education, and continuous performance in the entrepreneurial process.

Therefore, students’ entrepreneurial psychological quality should be cultivated through the integration of personal, family, school, and social factors. The development of the new media art environment provides a powerful development platform for the innovation and entrepreneurship of the students majoring in art design, enriches their entrepreneurial means, and offers them a powerful entrepreneurial channel (Lang and Liu, 2019). Higher education in colleges and universities makes important contributions to the development of Chinese society. With the requirements of the transformation and development of colleges and universities, the idea of running colleges and universities shifts to serving the local economy and cultivating applied technical talents. It is imperative to cultivate professional talents for editing and publishing in the publishing industry. The reasons include the decoupling of talent training in colleges and universities from the actual market demand, the single talent training mode, and the requirements of the digital transformation of the publishing industry (Wu et al., 2019). The traditional teaching mode and teaching method of entrepreneurship in colleges and universities are fixed, which is difficult to adapt to the development of new media art. Therefore, it is necessary to reform and innovate on the basis of the curriculum of art colleges and universities. The optimization of courses, the reform of teaching methods, and the application of innovative technologies in professional art colleges can improve the learning and entrepreneurial ability of students majoring in art design (Deng et al., 2021; Liu and Chen, 2021).

**Figure 1:**
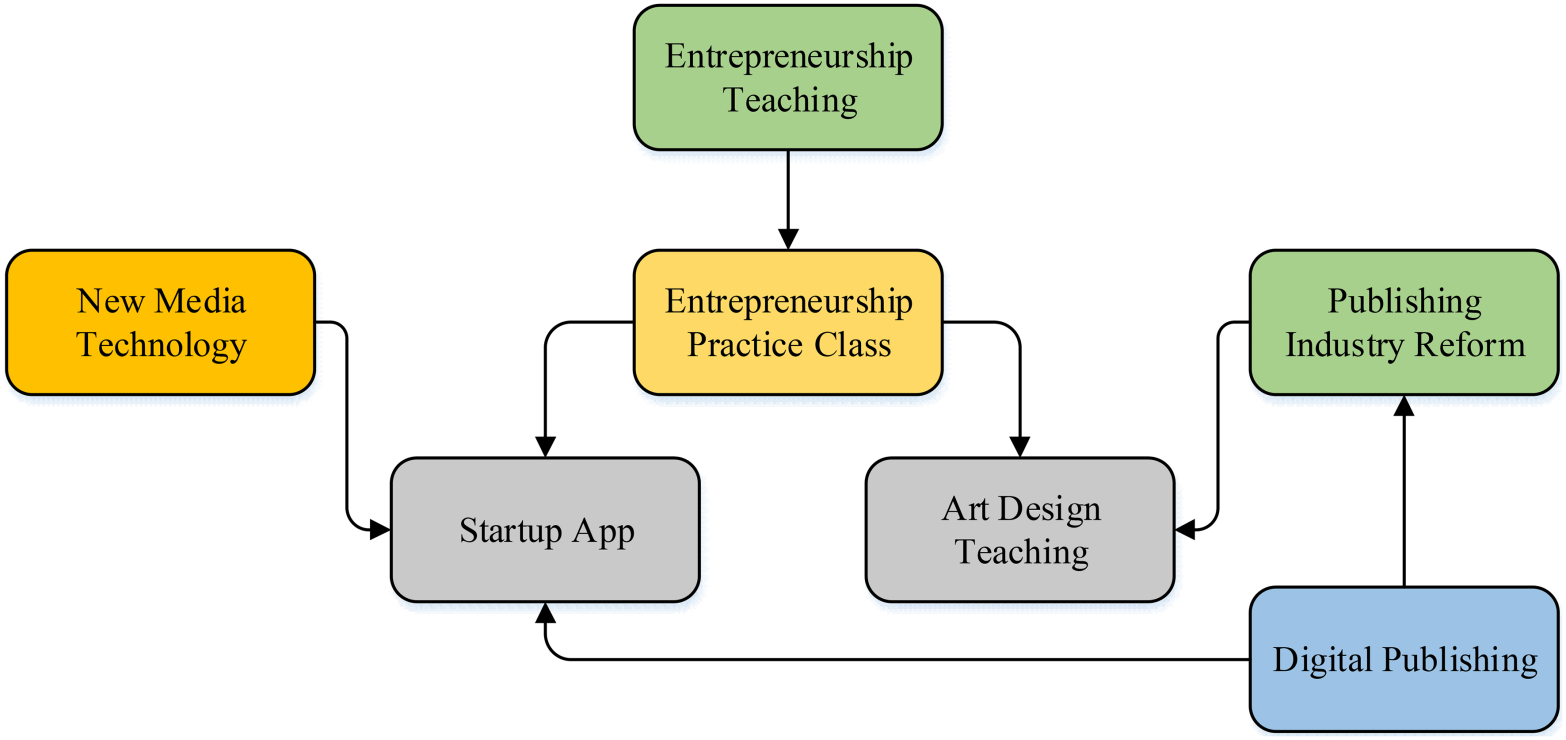
The relationship between students’ entrepreneurship and college teaching under the new media.

The traditional teaching mode in art colleges and universities is mainly based on knowledge imparting. Therefore, it is proposed to take the basic knowledge of other disciplines as an auxiliary, carry out cross-compound teaching, integrate more innovative knowledge into the curriculum of art students, and broaden students’ horizons and artistic ideas, improving the innovation ability of art students. Under the new media art, the course should combine Internet technology to change the traditional single classroom teaching mode and introduce network course teaching as a supplement to the course. While knowledge is imparted, a student learning group should be established, and it should have students from different majors. The teacher makes the members of the group discuss and analyze problems through step-by-step research, enriches the students’ perspective on problem analysis, and improves their organizational ability and discussion ability (Wu and Chen, 2021). Figure 1 presents the relationship between students’ entrepreneurship and college entrepreneurship teaching under the new media art.

### Questionnaire Design and Data Statistics

Under the new media art, the psychological states of college students majoring in art design need to be deeply studied and analyzed, and the study is carried out from two aspects of entrepreneurial interest and entrepreneurial activities to help students establish positive entrepreneurial psychological quality and enhance their innovative ability and entrepreneurial ability. In this study, college students from four full-time undergraduate colleges and universities in Shanghai are selected as the subjects. Three hundred and fifty students from grade one to grade four are selected to distribute the “questionnaire on the status quo of students’ entrepreneurial psychological states under the new media art.” The questionnaire is mainly divided into two parts: The first part is the basic information of college students’ entrepreneurship, including grade, gender, school, and specialty; the second part is the statistics of students’ entrepreneurial interest. Then, the Likert five scale is used as a measuring tool to calculate the score of the subject’s cognitive feelings quantitatively. The variables are mainly domestic and foreign relevant literature or the research questionnaire, and the relevant variables in the study are revised and improved by experts in the relevant research field. A total of 321 valid questionnaires are collected in the research process, and the recovery rate is 91.71%, which meets the requirements of data analysis.

SPSS 25.0 is used for statistical analysis of the collected questionnaire data, and confirmatory factor analysis is used to test the structural validity. And AVE (average variance extracted) method is used to test the difference validity, and Cronbach’s *α* coefficient is used to test the reliability of the scale. The distribution and recovery of questionnaires meet the needs of scientific research. To test the structural validity of the scale used, it is necessary to test the Kaiser-Meyer-Olkin (KMO) value. The KMO value of this scale is 0.967, and the KMO values of the other two potential variables are all above 0.7, indicating that the scale of this study has a good test effect and is suitable for factor analysis. The AVE method is used to analyze the discriminant validity of the scale. The calculation shows that the AVE values of each dimension of the scale are greater than the correlation coefficients between each dimension, so the scale used in this study has discriminant validity between each dimension. Cronbach’s *α* coefficient is used to process the collected data, and the reliability test of the total scale and the dimensions of the scale is carried out. The *α* coefficient of the overall dimensions is greater than the standard 0.7, indicating that the scale used has high internal consistency, good stability, and good overall reliability, which can meet the needs of scientific research.

## Statistical Data Analysis

### Analysis of Shanghai Publishing Industry

**Figure 2:**
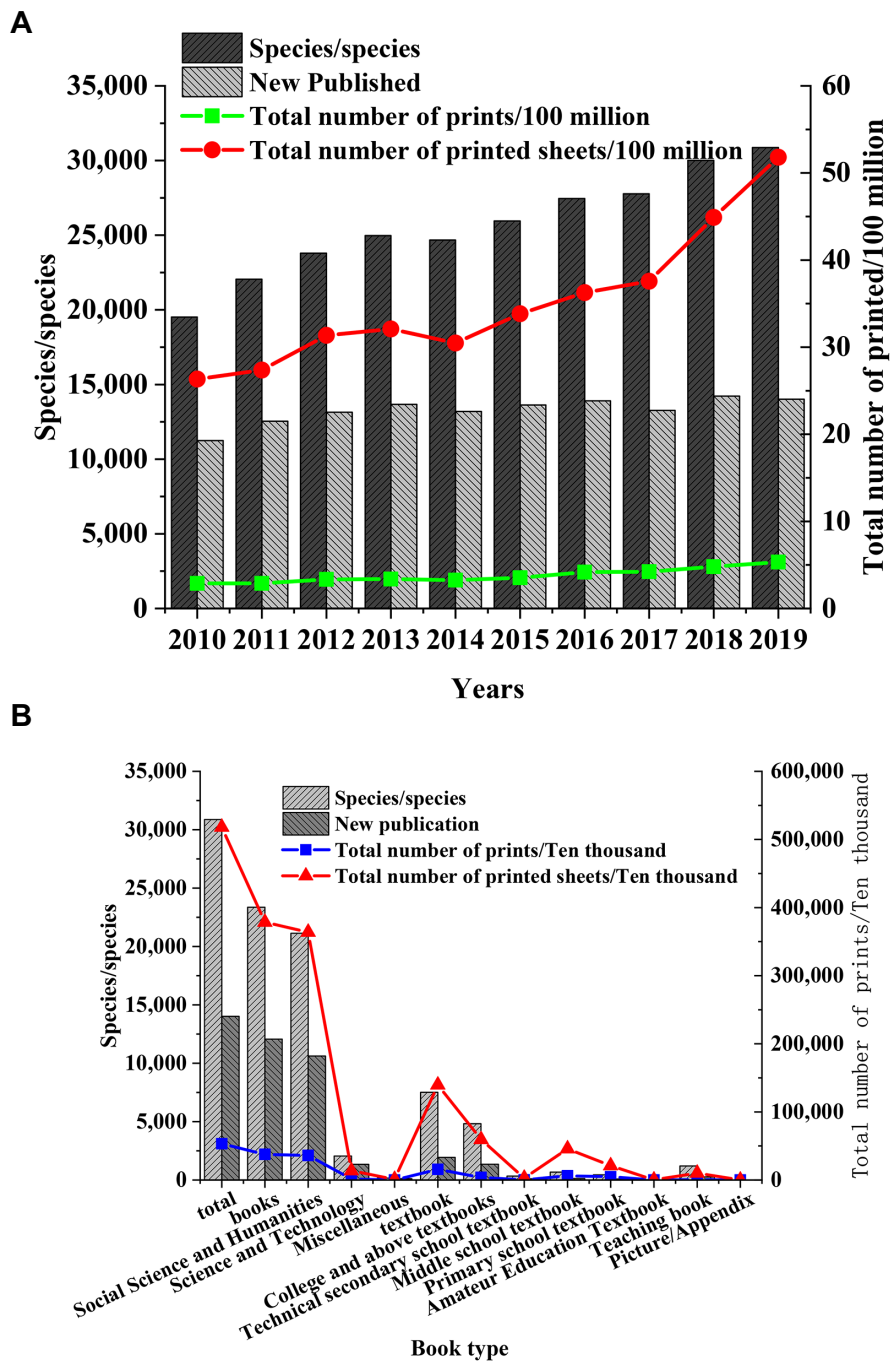
Number of books published in Shanghai. **(A)** Number of books published in Shanghai in 2010. **(B)** Types of books published in Shanghai in 2019.

**Figure 3:**
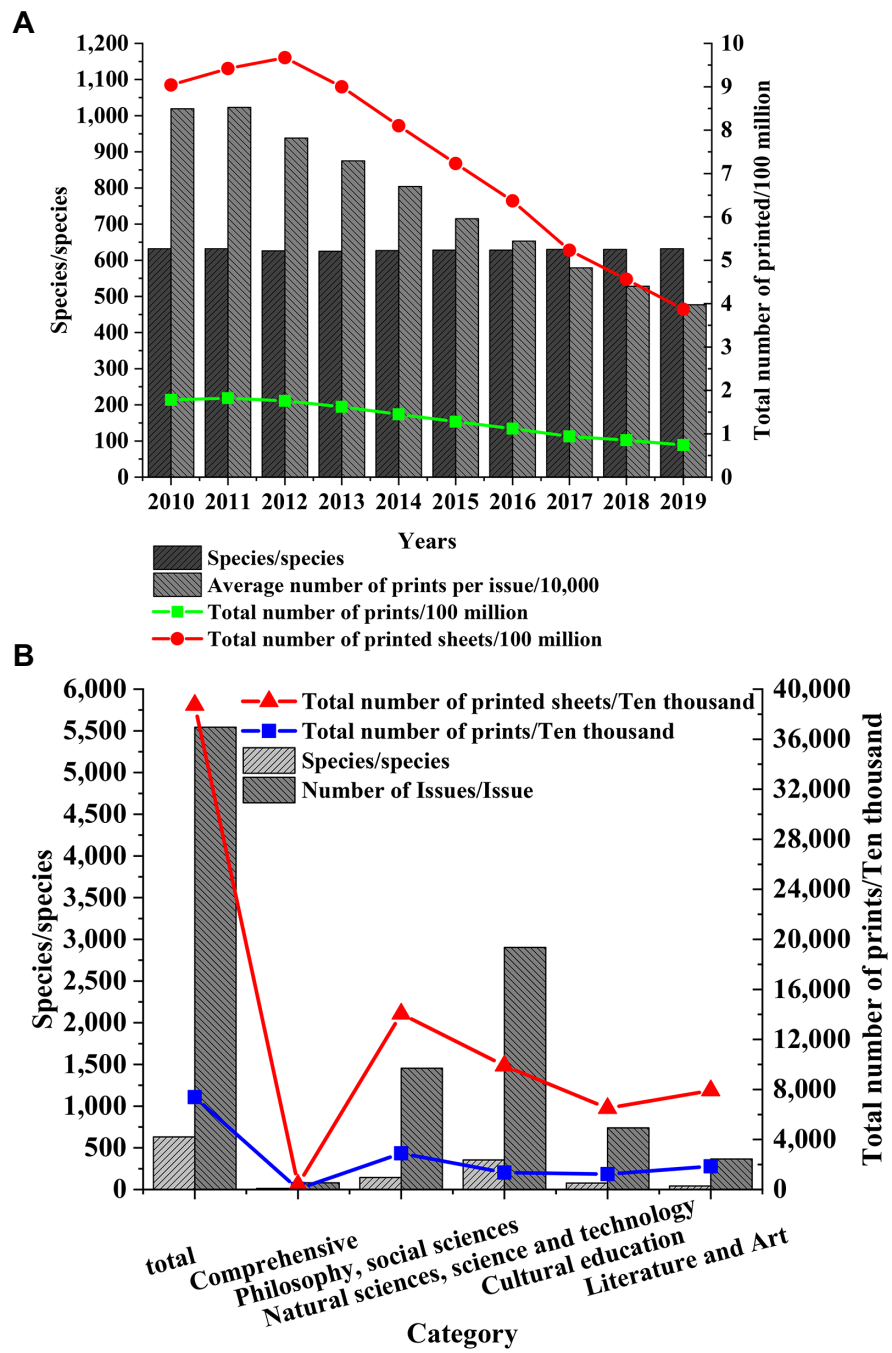
Number of journals published in Shanghai. **(A)** Number of Shanghai journals published in 2010. **(B)** Types of Shanghai journals published in 2019.

**Figure 4:**
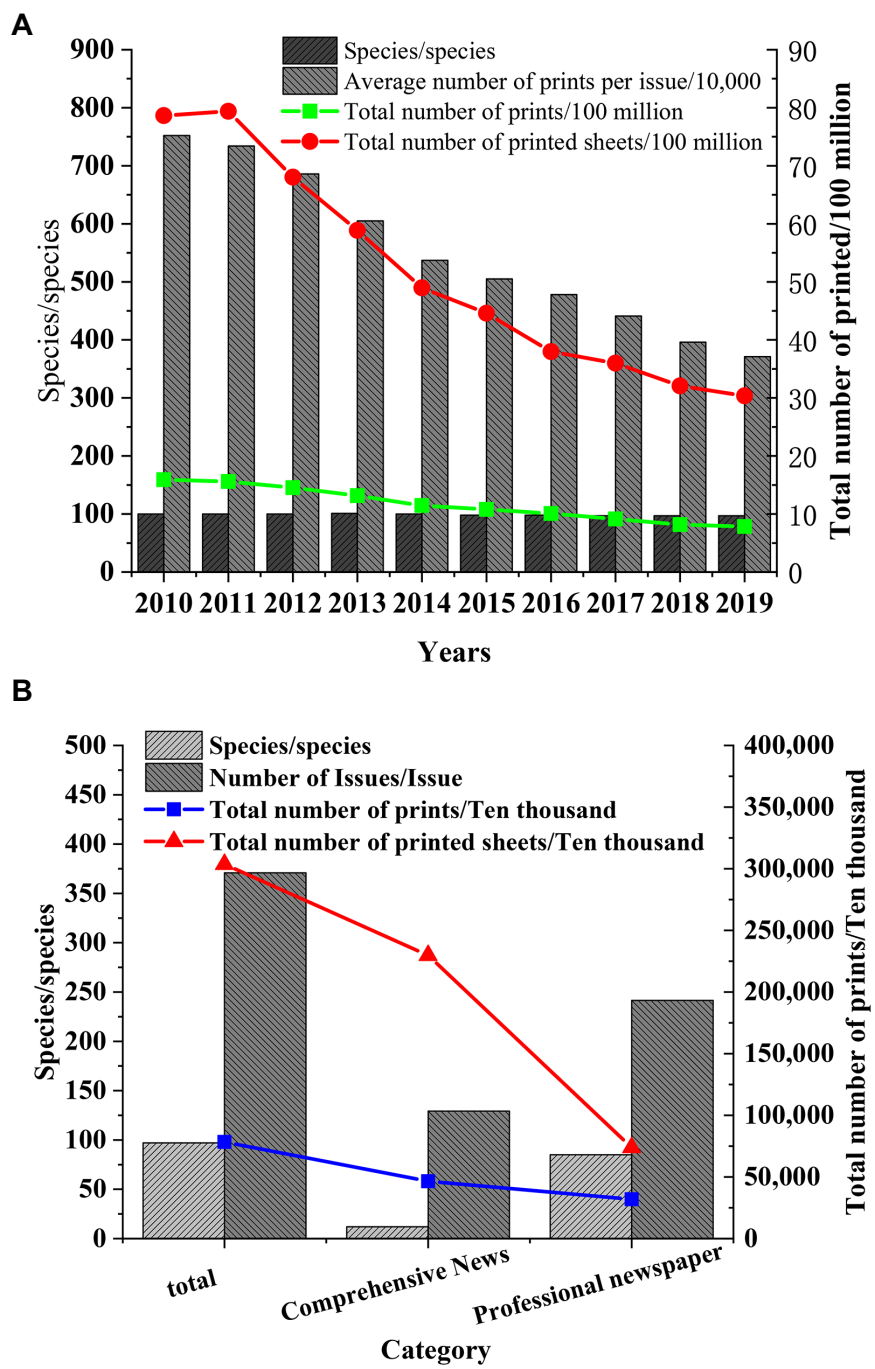
Number of Shanghai newspapers published. **(A)** Number of Shanghai newspapers published in 2010. **(B)** Types of Shanghai newspapers published in 2019.

Shanghai, the economic center of the Yangtze River Delta, is selected as the research object to study the development of the publishing industry in the Yangtze River Delta. After the data of the Shanghai Statistical Yearbook are collected, the changes in the number of books, periodicals, and newspapers in Shanghai in recent years are explored, and the status of the publishing industry in the Yangtze River Delta is speculated. The published data of books, periodicals, and newspapers from 2010 to 2019 are obtained, and the types of books, periodicals, and newspapers in Shanghai in 2019 are statistically analyzed (Figures 2–4).

Figure 2 shows that the number of the types of book publishing in Shanghai increase from 2010 to 2019, with about 13,000 new books published each year, and the total printing number increases year by year, indicating that the book marketers in Shanghai have great development potential. Among the books published in Shanghai in 2019, the number of books of social science, humanities, and textbooks is the largest, indicating that the residents in Shanghai have a large demand for humanistic books. Primary and secondary schools and various colleges and universities also have obvious needs for book publishing.

Figure 3 shows that the types of journals published in Shanghai remain unchanged, about 630. However, the average number of printed copies per period decreases year by year. The total number of printed copies and the total number of printed copies decreased sharply after 2012. In 2019, the total number of printed copies is 74 million and the total number of printed copies is 387 million. Among the journals published in Shanghai in 2019, the number of journals printed in philosophy and social science is the largest, which is 1.453 million, and the number of journals printed in natural science and technology is the largest, which is 354. Therefore, the overall demand for journals in Shanghai is declining year by year, and the competition in the journal market is more intense.

Figure 4 shows that the number of newspapers published in Shanghai begins to decline rapidly after 2011, and the types of newspapers remained unchanged. The average number per issue in 2019 decreases by nearly 50% compared with 2010. Newspaper is mainly divided into comprehensive newspapers and professional newspapers, and the total number of comprehensive newspapers is far more than that of professional newspapers.

In general, the Shanghai book publishing market still has great development potential, with humanities books and textbooks as the main publications. The demand for journals and newspapers is declining year by year, and the overall number of printing is also declining. The reason may be that under the development of new media, people can quickly obtain relevant information through the Internet, which greatly facilitates people’s access to information. The time and economic cost of obtaining information from journals are higher, and they have some disadvantages compared with digital publishing books.

### Result Analysis of the Questionnaire Survey

**Figure 5:**
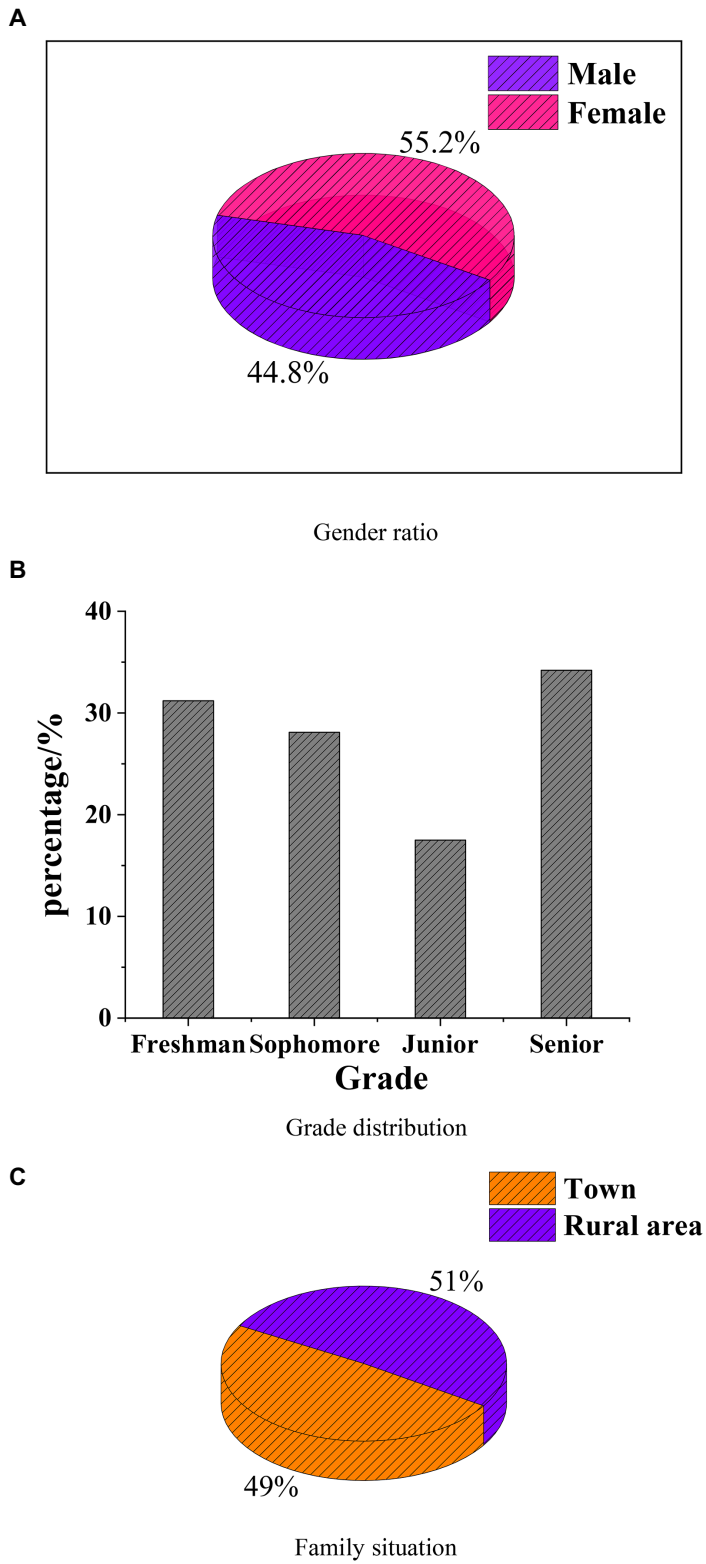
Basic information of the research object. **(A)** Gender ratio. **(B)** Grade distribution. **(C)** Family situation.

**Figure 6:**
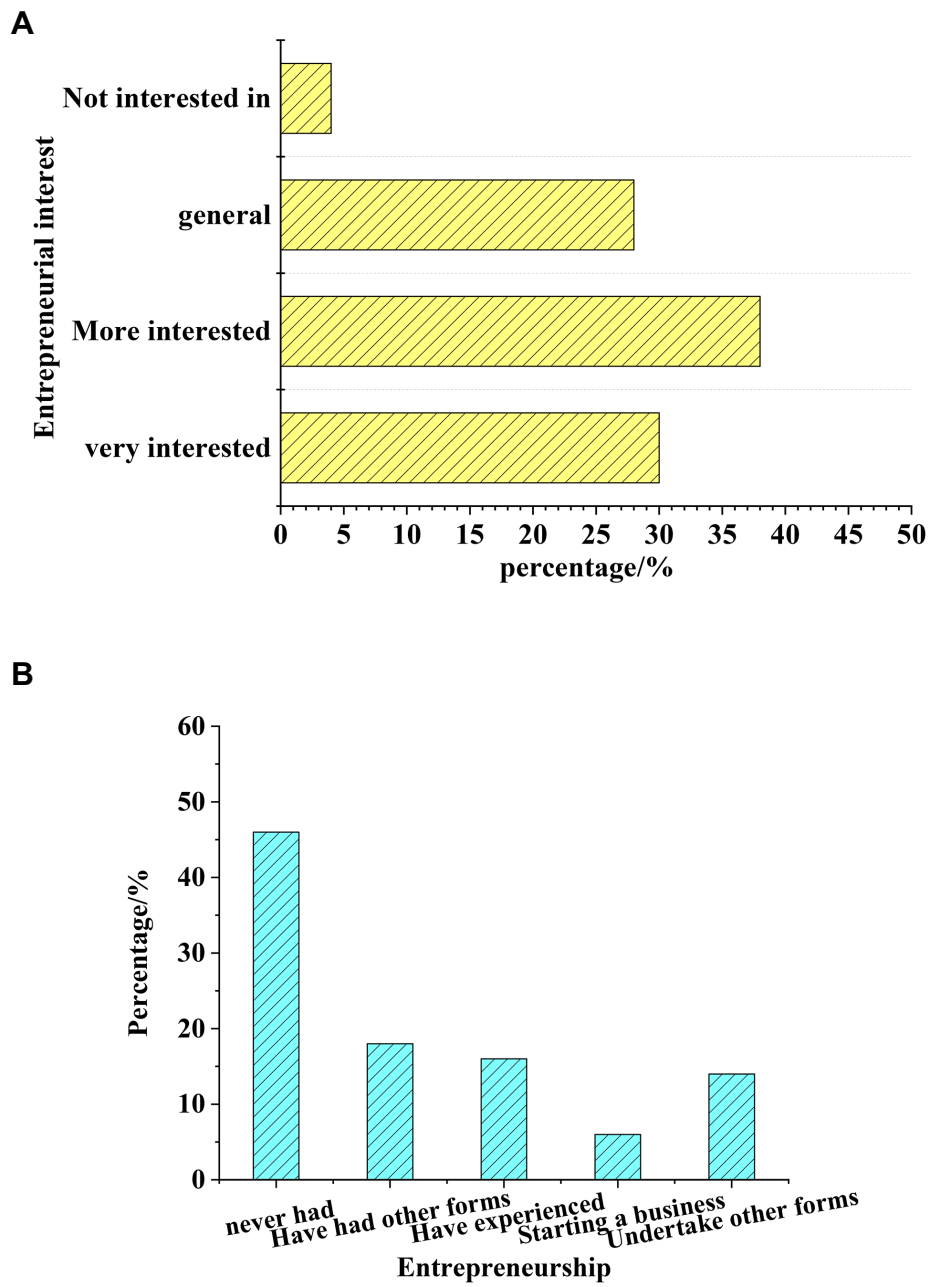
Statistics of the students’ entrepreneurship. **(A)** Statistics of their entrepreneurship interest. **(B)** Statistics of their entrepreneurship.

To study the interest and psychology of the students’ majoring in art design under the new media art, a statistical analysis of the questionnaire survey is made, and the students’ entrepreneurial interest and entrepreneurship are analyzed. The results are shown in Figures 5, 6.

Figure 5 shows that the proportion of girls is 55.2% and that of boys is 44.8%. The grades of students are freshmen, sophomores, and seniors, among whom freshmen and seniors have the largest number of subjects, which are 31.2 and 34.2%, respectively. 51% of the subjects are from urban areas and 49% from rural areas, and the proportion is the same. The results of the questionnaire show that the number of boys and girls in cities is the same as that in rural areas, and the number of freshmen and seniors pay more attention to entrepreneurship. The reason may be that students who just enter universities or will graduate are more concerned with their future development, so they also pay more attention to entrepreneurship.

Figure 6 shows that 68% of the students are very interested in entrepreneurship, 28% are not enthusiastic about entrepreneurship, and 4% are completely not interested in entrepreneurship. The statistical analysis of students’ entrepreneurship shows that 53% of students have not carried out entrepreneurship activities at all, and 19% are carrying out entrepreneurship activities. This shows that students have good enthusiasm for entrepreneurship in the new media environment, but most students have not carried out entrepreneurial activities due to the influence of their entrepreneurial ability and policies.

In the new media environment, the development of the publishing industry in Shanghai is limited. The development of digital media affects the number of journals and newspapers published. Therefore, the publishing industry is required to carry out digital reform and enhance the competitiveness of journals and newspapers in the region through the integration of traditional publishing and digital publishing. Shanghai, as the economic center of the Yangtze River Delta region, the development of Shanghai’s publishing industry also directly affects the process of regional publishing industry integration. Therefore, it can be speculated that the development of the publishing industry in the Yangtze River Delta region is also facing similar problems. Under this circumstance, it is required to reform the publishing industry in the region and enhance the competitiveness of the industry through innovative ways, Therefore, in view of this problem, art colleges and universities should optimize the curriculum structure and teaching mode of entrepreneurship education, cultivate students’ ability of innovation and entrepreneurship, enhance students’ entrepreneurial ability in the new media environment, adapting to the needs of social and economic development, and publishing industry reform and achieving successful entrepreneurship.

## Conclusion

The study aims to explore the development status and reform direction of the publishing industry under the development of new media and to provide strong talent support for the development of the industry. The study discusses the development status of the publishing industry in Shanghai, the economic center of the Yangtze River Delta region, and analyzes the problems existing in the development. First of all, it is proposed to improve the development and competitiveness of the publishing industry in the region through resource allocation. Second, use questionnaire surveys to collect the entrepreneurial willingness and activity status of students in art colleges and universities, and study students’ entrepreneurial psychology and entrepreneurial ability. The results reveal that Shanghai’s books, periodicals, and newspapers are the main publishing areas, among which books have good development potential, but the development of the new media industry has had a serious impact on the development of periodicals and newspapers, so the publishing industry is required to reform. The students in this area have a strong interest in entrepreneurship, but due to their own lack of innovation and entrepreneurship capabilities, most students have not carried out entrepreneurial activities. Therefore, it is necessary for students to have a positive entrepreneurial psychology to optimize the teaching model and enhance the innovation and entrepreneurship ability of students in relevant schools.

However, there are still some shortcomings. It has not conducted a field investigation on the development status of publishing industry in the Yangtze River Delta region and cannot accurately reflect the current research status. Concentrated in a certain area, it does not reflect the situation of student entrepreneurship in most areas. Therefore, in the follow-up research, field research will be carried out, and the questionnaire will be designed in more detail to effectively reflect the current situation of the publishing industry in the Yangtze River Delta region and the actual situation of student entrepreneurship and expand the sample of the research area. The study provides a reference for studying the entrepreneurial situation and publishing industry status of art and design college students in the Yangtze River Delta.

## Data Availability Statement

The original contributions presented in the study are included in the article/supplementary material, further inquiries can be directed to the corresponding author.

## Ethics Statement

The studies involving human participants were reviewed and approved by Nanjing University Ethics Committee. The patients/participants provided their written informed consent to participate in this study. Written informed consent was obtained from the individual(s) for the publication of any potentially identifiable images or data included in this article.

## Author Contributions

MZ: writing - draft, software, and data curation. FS: conceptualization, methodology, resources, and supervision. All authors contributed to the article and approved the submitted version.

## Conflict of Interest

The authors declare that the research was conducted in the absence of any commercial or financial relationships that could be construed as a potential conflict of interest.

## Publisher’s Note

All claims expressed in this article are solely those of the authors and do not necessarily represent those of their affiliated organizations, or those of the publisher, the editors and the reviewers. Any product that may be evaluated in this article, or claim that may be made by its manufacturer, is not guaranteed or endorsed by the publisher.

## References

[ref1] Augustyn K. (2020). The global book publishing market as an interdisciplinary research field. Z. Inf. Nauko. Stud. Inf. 58, 122–146. doi: 10.36702/zin.728

[ref33] Boratyńska K. (2019). Impact of digital transformation on value creation in Fintech services: an innovative approach. J. Promotion. Manage. 25, 631–639. doi: 10.1080/10496491.2019.1585543

[ref2] Cai W. Lysova E. I. Khapova S. N. Bossink B. A. (2019). Does entrepreneurial leadership foster creativity among employees and teams? The mediating role of creative efficacy beliefs. J. Bus. Psychol. 34, 203–217. doi: 10.1007/s10869-018-9536-y

[ref3] Cele L. Wale E. (2020). Determinants of smallholders’ entrepreneurial drive, willingness and ability to expand farming operations in KwaZulu-Natal. Dev. Pract. 30, 1028–1042. doi: 10.1080/09614524.2020.1764501

[ref34] Çöteli S. (2019). The impact of new media on the forms of culture: digital identity and digital culture. Online. J. Commun. Medi. 9:e201911. doi: 10.29333/ojcmt/5765

[ref4] Deng X. Guo X. Wu Y. J. Chen M. (2021). Perceived environmental dynamism promotes entrepreneurial team member’s innovation: explanations based on the uncertainty reduction theory. Int. J. Environ. Res. Public Health 18:2033. doi: 10.3390/ijerph18042033, PMID: 33669732 PMC7921965

[ref5] Fan J. (2019). The publishing industry in new China: eventful 7 decades. Publ. Res. Q. 35, 629–647. doi: 10.1007/s12109-019-09694-0

[ref6] Fang Y. C. Chen J. Y. Wang M. J. Chen C. Y. (2019). The impact of inclusive leadership on employees’ innovative behaviors: the mediation of psychological capital. Front. Psychol. 10:1803. doi: 10.3389/fpsyg.2019.01803, PMID: 31447740 PMC6691172

[ref7] Feng B. Chen M. (2020). The impact of entrepreneurial passion on psychology and behavior of entrepreneurs. Front. Psychol. 11:1733. doi: 10.3389/fpsyg.2020.01733, PMID: 32793066 PMC7385187

[ref8] Ferreira J. Coelho A. Moutinho L. (2020). Dynamic capabilities, creativity and innovation capability and their impact on competitive advantage and firm performance: the moderating role of entrepreneurial orientation. Technovation 92:102061. doi: 10.1016/j.technovation.2018.11.004

[ref9] Genc E. Dayan M. Genc O. F. (2019). The impact of SME internationalization on innovation: the mediating role of market and entrepreneurial orientation. Ind. Mark. Manag. 82, 253–264. doi: 10.1016/j.indmarman.2019.01.008

[ref10] Girija S. (2019). Political economy of media entrepreneurship: commercialization and commodification in a digital news media enterprise. J. Media Manag. Entrep. 1, 27–39. doi: 10.4018/JMME.2019010102

[ref11] Girija S. (2020). Political economy of media entrepreneurship: power, control and ideology in a news media enterprise. Nord. J. Media Manag. 1, 81–101. doi: 10.5278/njmm.2597-0445.3651

[ref12] Have I. Pedersen B. S. (2020). The audiobook circuit in digital publishing: V. Sil. Revol. New Media Soc. 22, 409–428. doi: 10.1177/1461444819863407

[ref13] Hwang W. S. Choi H. Shin J. (2020). A mediating role of innovation capability between entrepreneurial competencies and competitive advantage. Technol. Anal. Strateg. Manag. 32, 1–14. doi: 10.1080/09537325.2019.1632430

[ref14] Irfanullah H. M. (2021). So, what does resilience mean for scholarly publishing. Learn. Publ. 34, 57–63. doi: 10.1002/leap.1351

[ref15] Lang C. Liu C. (2019). The entrepreneurial motivations, cognitive factors, and barriers to become a fashion entrepreneur: a direction to curriculum development for fashion entrepreneurship education. Int. J. Fashion Des. Technol. Educ. 12, 235–246. doi: 10.1080/17543266.2019.1581844

[ref16] Liu Y. Chen M. (2021). Applying text similarity algorithm to analyze the triangular citation behavior of scientists. Appl. Soft Comput. 107:107362. doi: 10.1016/j.asoc.2021.107362

[ref17] Magadán-Díaz M. Rivas-García J. (2019). Crowdfunding in the Spanish publishing industry. Publ. Res. Q. 35, 187–200. doi: 10.1007/s12109-019-09643-x

[ref18] Magadán-Díaz M. Rivas-García J. (2020a). A transaction cost economics view on outsourcing decision in Spanish publishing industry. Nord. J. Media Manag. 1, 235–260. doi: 10.5278/njmm.2597-0445.5422

[ref19] Magadán-Díaz M. Rivas-García J. I. (2020b). The publishing industry in Spain: a perspective review of two decades transformation. Publ. Res. Q. 36, 335–349. doi: 10.1007/s12109-020-09746-w

[ref20] Magadán-Díaz M. Rivas-García J. (2021). The role of KIBS on digital transformation and technological changes in the Spanish publishing industry. Malays. J. Libr. Inf. Sci. 26, 83–101.

[ref21] Ritter T. Pedersen C. L. (2020). Digitization capability and the digitalization of business models in business-to-business firms: past, present, and future. Ind. Mark. Manag. 86, 180–190. doi: 10.1016/j.indmarman.2019.11.019

[ref22] Sandberg J. Holmström J. Lyytinen K. (2020). Digitization and phase transitions in platform organizing logics: evidence from the process automation industry. MIS Q. 44, 129–153. doi: 10.25300/MISQ/2020/14520

[ref23] Sirén C. Parida V. Frishammar J. Wincent J. (2020). Time and time-based organizing of innovation: influence of temporality on entrepreneurial firms’ performance. J. Bus. Res. 112, 23–32. doi: 10.1016/j.jbusres.2020.02.028

[ref24] Toscher B. (2019). Entrepreneurial learning in arts entrepreneurship education: a conceptual framework. Artivate 8, 3–22.

[ref25] Toscher B. Dahle Y. Steinert M. (2020). Get Give Make Live: an empirical comparative study of motivations for technology, youth and arts entrepreneurship. Soc. Enterp. J. 16, 179–202. doi: 10.1108/SEJ-03-2019-0016

[ref26] Watson R. (2019). Predatory journals and the pollution of academic publishing. J. Nurs. Manag. 27, 223–224. doi: 10.1111/jonm.12739, PMID: 30506601

[ref27] Wei J. Chen Y. Zhang Y. Zhang J. (2020). How does entrepreneurial self-efficacy influence innovation behavior? Exploring the mechanism of job satisfaction and Zhongyong thinking. Front. Psychol. 11:708. doi: 10.3389/fpsyg.2020.00708, PMID: 32457676 PMC7227373

[ref28] Wei X. Liu X. Sha J. (2019). How does the entrepreneurship education influence the students’ innovation? Testing on the multiple mediation model. Front. Psychol. 10:1557. doi: 10.3389/fpsyg.2019.01557, PMID: 31354574 PMC6636545

[ref29] Wibisono A. W. B. Koesrindartoto D. P. (2020). Business strategy formulation for publishing company (case study: ABC Press). Eur. J. Bus. Manag. Res. 5:535. doi: 10.24018/ejbmr.2020.5.5.535

[ref30] Wu Y. J. Chen J.-C. (2021). Stimulating innovation with an innovative curriculum: a curriculum design for a course on new product development. Int. J. Manag. Educ. 19:100561. doi: 10.1016/j.ijme.2021.100561

[ref31] Wu Y. J. Chen S. C. Pan C. I. (2019). Entrepreneurship in the internet age: internet, entrepreneurs, and capital resources. Int. J. Semant. Web Inf. Syst. 15, 21–30. doi: 10.4018/IJSWIS.2019100102

[ref32] Wu Y. C. J. Wu T. (2017). A decade of entrepreneurship education in the Asia Pacific for future directions in theory and practice. Manag. Decis. 55, 1333–1350. doi: 10.1108/MD-05-2017-0518

